# The Prevalence, Patterns and Correlates of Childhood Trauma Exposure in a Nationally Representative Sample of Young People in Northern Ireland

**DOI:** 10.1007/s40653-022-00449-2

**Published:** 2022-05-07

**Authors:** Enya Redican, Jamie Murphy, Orla McBride, Lisa Bunting, Mark Shevlin

**Affiliations:** 1 School of Psychology, Ulster University, Coleraine, UK; 2 Queen’s University, Belfast, UK

**Keywords:** Childhood trauma, Northern Ireland, Latent class analysis, Adolescents

## Abstract

**Purpose:**

Childhood trauma (CT) exposure is common, with many young people affected by multiple co-occurring traumas.

**Methods:**

Participants were a representative sample of 11–19-year-olds (n = 1293), who participated in the largest ever representative survey of youth mental health in Northern Ireland (NI) – the NI Youth Wellbeing Prevalence Survey 2020. This study used latent class analysis (LCA) to identify typologies that were most representative of trauma experience and co-occurrence among young people living in NI. Demographic, parental and deprivation variables were then used within a multinomial logistic regression analysis to describe trauma class membership.

**Results:**

Over 35% (n = 478) of participants reported exposure to at least one CT, with over 50% (n = 259) of trauma-exposed young people reporting multiple trauma exposure. LCA results provided support for a three-class model; ‘low-exposure’, ‘moderate-exposure: community-victimization’ and ‘high-exposure: sexual-trauma’. While none of the child, parental or familial covariates differentiated members of the ‘moderate-exposure: community-victimization’ from ‘low-exposure’, those in ‘high-exposure: sexual-trauma’ were over four and a half times more likely to belong to a family in receipt of income benefits and over ten times more likely to have experienced some form of out-of-home care.

**Conclusions:**

This study highlights the presence of three distinct trauma classes in the NI adolescent population. In particular, this study identifies a small minority of young people who have experienced multiple CT’s, including sexually based traumas, with these traumas most likely to have occurred in the context of out-of-home care and familial poverty.

## Introduction

Trauma is defined as *‘exposure to an extremely threatening or horrific event or series of events’* (ICD-11, 2018). While evidence indicates that trauma, is highly prevalent among young people, e.g. approximately half of children and adolescents (hereafter ‘young people’ unless otherwise specified) have been shown to be exposed to a traumatic event prior to reaching adulthood (Smith et al., 2019), there is a paucity of nationally representative studies and data to adequately inform our understanding of CT prevalence. This is problematic given that these samples afford a more comprehensive understanding of CT prevalence and thus, have relevant implications for practitioners, researchers and policy-makers alike (Saunders & Adams, 2014).

Where nationally representative studies of CT prevalence among young people have been conducted, they have indicated CT exposure to be common. A recent epidemiological survey of young people living in England and Wales revealed that 31.1% of young people in the general population were trauma-exposed (Lewis et al., 2019). In a nationally representative sample of young people in Switzerland, 56.1% of young people were identified as trauma-exposed (Landolt et al., 2013), whilst in the United States, CT prevalence was 61.9% among 13–17 year olds (McLaughlin et al., 2013) and 67.8% among those aged 9–16 years (Copeland et al., 2007).

There are no representative studies of CT prevalence among young people living in Northern Ireland (NI). Prior studies that have utilised representative data to understand the prevalence of CT exposure in NI have largely focused on prevalence of exposure to adverse childhood experiences (ACEs) derived from adult retrospective recall. For instance, as part of the Multiple Adverse Childhood Experiences (MACE) research project, Mc Gavock and Spratt (2014) found that 56% of university students in NI had experienced at least one ACE during their lifetime. Utilising data from the NI Study of Health and Stress (NISHS), McLafferty et al. (2016) found that 32% of adults in NI were exposed to one or more ACEs. However, given that adult recall of childhood trauma and adversity has been shown to be vulnerable to bias and error (Colman et al., 2016; Reuben et al., 2016), and that extant studies have focussed on childhood adversities rather than clinically defined traumatic experiences, determining the prevalence of CT exposure in young people in NI using representative data is an important research endeavour. This is especially pertinent given the legacy of the “Troubles”, a colloquial term used to describe a thirty-year long period (1969–1999) of violent conflict in NI in which exposure to riots, shootings, bombings, kidnappings as well as a host of other traumatic events were a feature of daily life (Ferry et al., 2014). It is postulated that the transgenerational transmission of trauma stemming from the “Troubles”, may continue to adversely affect young people in NI today (O’Neill et al., 2015). Intergenerational trauma describes how the effects of trauma exposures can transmit across generations (Yehuda & Lehrner, 2018), with factors such as the post-traumatic symptoms of the parent (Narayan et al., 2021; Lünnemann et al., 2019), parenting style and compromised parent-child interactions (e.g. Schwerdtfeger et al., 2013; Kaitz et al., 2009), as well as epigenetic mechanisms (Yehuda & Lehrner, 2018), postulated to explain this phenomenon. Thus, within the NI context, pathways through which the NI “Troubles” may continue to affect young people include epigenetic risks from parental mental health difficulties, and wider economic and social post-conflict factors, to name a few (O’Neill et al., 2015).

Despite the recognition that CTs frequently co-occur (e.g., Finkelhor et al., 2005, 2007a, 2009; Houston et al., 2011; Shevlin & Elklit, 2008), often across multiple trauma categories (e.g. Finkelhor et al., 2007a) – a concept typically referred to as ‘poly-victimisation’, the most commonly used analytic approaches to quantifying multiple traumatic exposure, despite being highly useful, are unlikely to capture this particular phenomenon. The vast majority of studies to date have (1) used a single trauma variable as a predictor (e.g. Shin et al., 2010), (2) used multiple trauma variables simultaneously as predictors (e.g. Cecil et al., 2017; Vacek & Whisman, 2020), or (3) a count (summed) score to represent multiple CT exposure (e.g. Cloitre et al., 2009; Rasmussen et al., 2020). These approaches are considered ‘variable-centred’, where the focus is on trauma exposure (Houston et al., 2011; Shevlin & Elklit, 2008), whereas latent class analysis (LCA) shifts the level of analysis to the individual and has been supported as the optimal method to model trauma exposure patterns (O’Donnell et al., 2017). LCA is a type of a mixture model which classifies individuals into homogenous groups, or classes, based on similar response patterns to observed categorical indicators (Nylund et al., 2007). The advantages of LCA are that it allows for (1) the identification of distinct subgroups of individuals with the same trauma-exposure patterns, (2) identification of risk-factors specific to each trauma sub-group, and (3) the assessment of differential associations between trauma exposure sub-groups and mental health outcomes (Finkelhor et al., 2007; Houston et al., 2011; Jenness & McLaughlin, 2015; O’Donnell et al., 2017).

In their systematic review of all studies which applied LCA to understanding trauma exposure patterns, O’Donnell et al. (2017) reported how (1) most studies identified four qualitatively distinct trauma groups comprising of individuals with the same trauma exposure profile, (2) trauma classes were distinguishable on the basis of CT exposure probability and the presence/absence of a sexual-trauma class, and (3) the identified trauma classes were differentially associated with psychological outcomes. Despite the utility of LCA to understanding CT exposure patterns, no such study has been conducted in NI utilising a large nationally representative sample of young people. Only one study by MacLochlainn et al. (2021) applied LCA to determine profiles of stressful life events, but not traumatic stressors, in an adolescent sample in NI. To facilitate the development of generalizable prevention and intervention strategies, examining trauma classes across different countries and cultural contexts is crucial (Charak et al., 2020), especially given the unique social and political context of NI where exposure to violence has been observed to be high in young people (McAloney et al., 2009). Thus, determining classes of CT exposure in young people living in NI will elucidate the various patterns of CT exposure across the general youth population.

Identifying factors which predict and distinguish trauma groups is an important step in the development of precise prevention and intervention programmes (Adams et al., 2016). To date, risk factors identified for membership of trauma classes characterised by high levels of exposure to a variety of traumatic experiences include living with less than both biological parents (McChesney et al., 2015; Finkelhor et al., 2011; Shevlin & Elklit, 2008), older age and parental educational attainments (Liang et al., 2020), economic adversity (e.g. McLaughlin et al., 2013), experiences of out-of-home care (i.e. periods in which the child was not living in the familial home) and ethnicity (Adams et al., 2016). No studies (to the best of the author’s knowledge) have investigated the influence of parental factors in differentiating trauma profiles. This is problematic given that parental psychopathology is linked to increased risk of trauma in young people (Koenen et al., 2010) whilst the intergenerational effects of parental adverse childhood experiences (ACEs) on offspring mental health are well-documented (e.g. Doi et al., 2020).

Consequently, the present study had several aims. The first aim was to determine the prevalence of trauma-exposure in young people aged 11–19 years in NI. It was hypothesized that findings would be largely consistent with other large-scale general-population investigations of young people and in particular with those observed in England and Wales (i.e. Lewis et al., 2019). The second aim was to determine gender differences in CT endorsement. It was hypothesized that females would report greater exposure to traumas considered as ‘high impact’ such as sexual-related traumas whilst males would report higher levels of exposure to non-interpersonal violent traumas (Tolin & Foa, 2008). The third aim was to identify groups of young people characterised by the same patterns of trauma exposure, whilst statistically adjusting for the potential role of gender. It was hypothesized that at least two trauma groups would be identified; a group characterised by high levels of traumatic exposure and another characterised by low levels and that gender would distinguish the identified trauma groups (O’Donnell et al., 2017). Although much is known surrounding the types of traumatic events which are likely to cluster and co-occur, due to the lack of research on samples of young people and the heterogeneity in the methodological procedures employed by those studies, an exploratory rather than confirmatory LCA approach was deemed most suitable for the present study. The final aim of the study was to identify those child, familial and parental factors that uniquely predicted trauma group membership. Based on previous research, it was expected that both child (i.e. gender, older age, ethnicity, out-of-home experiences) and familial factors (i.e. household composition, socioeconomic status, parental education) would predict membership to more severe CT groups however, because no study has examined parental predictors, no a- priori hypotheses were made in this regard.

## Methods

### Participants

Data for the current study was obtained from the Northern Ireland Youth Wellbeing Prevalence Survey (YWS-NI, 2020; see Bunting et al., 2020) which sought to determine the prevalence of mental health problems such as mood and anxiety disorders, post-traumatic stress disorder and complex post-traumatic stress disorder, in a nationally representative sample of young people aged 2 to 19 years in NI. Data collection took place between June 2019 and March 2020, prior to the COVID-19 Pandemic lockdowns. Participants were randomly recruited via the pointer database, a postcode register of all households in NI. A total of 21,730 addresses were drawn, of which 79% were deemed ineligible on the basis of no young person residing at the address (83%), the resident status of the addresses being unconfirmed (9%), or addresses being vacant or not able to be found (7%). Of the eligible households (n = 4,621), 67% participated, with reasons for non-participation including refusals (32%), and unavailability for participation (1%). This resulted in a final total of 3,074 parents or young person surveys being completed for the mental health component of the survey, and 2,815 parent surveys being completed. For the current study, only participants aged 11–19 years (n = 1299) were included in the analyses. Young people aged 11–19 years completed their own survey, with consent required from both the parent and young person aged 11–15 years, and the young person only if aged 16–19 years. Parent questionnaires were also completed, with the exception for those aged 16–19 years who were no longer residing in the caregiving home. For young people aged 16–19 years living in the parental home who did not wish for their parent/guardian to participate or the parent/guardian refused to participate, the young person was asked additional demographic questions. The purpose of the parent survey was to allow exploration of the associations between youth wellbeing and parental health and psychological wellbeing, and environmental factors (Bunting et al., 2020). Ethical approval for the current study was obtained from the research ethics committee at Ulster University.

Gender was dichotomized as (male = 0, female = 1) and thus, participants (n = 2) who identified as ‘other’ or where there was missing data on the gender variable (n = 4), were excluded from the study. The final sample included a total of 1,293 participants, of which the ratio of males to females was relatively equal (male = 51.2% (n = 662); female = 48.8% (n = 631)). The sample characteristics are reported in Table 1.

**Table 1:** Sociodemographic Characteristics of sample (N = 1293)

	% (n)
**Sex**	
Male	51.2 (662)
Female	48.8 (631)
**Age in years**	
11–15	51.7 (669)
16–19	48.3 (624)
	M = 15.15, SD = 2.582
**Child ethnicity**	
White	95.6 (1235)
Non-white	4.4 (57)
**Special educational needs**	
No	85.5 (1059)
Yes	14.5 (179)
**Out-of-home care**	
Yes	3.4 (44)
No	96.6 (1249)
**Highest household educational attainment**	
GCSE or below	32.9 (505)
A-levels or above	60.9 (788)
**Highest household employment level**	
Unemployed	14.6 (187)
At least one parent employed	85.4 (1092)
**Household composition**	
Not living with both biological parents	35.8 (460)
Living with both biological parents	63.8 (825)
**Family in receipt of income disability benefits**	
No benefits	64.1 (829)
Receives benefits	35.9 (464)
**Parent Mental Health**	
No problems	80.1 (1036)
Mental Health Problem	19.9 (257)
**Parent ACEs**	
Low ACE score	89.6 (1158)
High ACE score	10.4 (135)
**Area level deprivation**	
1 – most deprived	19.1 (247)
2	18.4 (238)
3	20.0 (259)
4	20.8 (269)
5 – least deprived	21.7 (280)

### Measures

#### Trauma Exposure

The traumatic events checklist, a 14-item checklist which forms part of the Child and Adolescent Trauma Screen (CATS; Sachser et al., 2017), was used to assess participants’ exposure to CTs. It was developed to screen for potentially traumatic events that meet Criterion-A in DSM-5 (American Psychiatric Association [APA], 2013). The CATS includes items which tap into interpersonal (e.g., experiencing or witnessing violence in the school or in the community, experiencing or witnessing violence at home, online sexual harassment, sexual assault, sexual molestation) and non-interpersonal (e.g., serious accident or injury) traumas. Items are scored dichotomously as yes (1) or no (0) responses. The CATS has been used to screen for trauma in the community (Kazlauskas et al., 2020) and trauma-exposed samples (Bruckmann et al., 2020). The CATS trauma screen has been shown to possess good test re-test reliability, convergent validity, and criterion validity across a number of countries and cultural contexts (e.g., Sachser et al., 2017; Dowdy-Hazlett et al., 2021; Nilsson et al., 2021).

### Predictor variables

**Child variables**: Child variables include gender (male = 0, female = 1), age (measured in years), ethnicity (non-white = 0, white = 1), out-of-home care (no = 0, 1 = yes) and special educational needs (no = 0, yes = 1). Out-of-home care describes experiences of living away from the caregiving environment and was assessed by asking young people “have you ever lived away from home…”. Out-of-home care was positively endorsed if an individual responded ‘yes’ to any of the following items: spending time in (a) a children’s home, (b) with non-relative foster parents, (c) with kinship carers (placement with family members or friends, arranged by social worker), (d) with kinship carers (living with family members other than parents or friends with no social work involvement, (e) in a secure accommodation or a juvenile justice unit or (f) other experience of living away from home. Special educational needs was assessed by asking parents of 11–15 year olds ‘*does your child have a diagnosed or suspected special educational need?’* and by asking 16–19 year olds *‘while at school, did you ever have a diagnosed or suspected special educational need’.* Items were coded as no (0) and 1 (yes).

#### Family variables

Family variables included highest household education attainment (GCSE or below (secondary school qualification typically taken by 14–16 year olds in the UK) = 0, A-levels or above (secondary school qualification typically taken by 16–18 year olds in the UK) = 1), highest household employment status (unemployed = 0, at least one parent employed = 1), household composition (not living with both biological parents = 0, living with both biological parents = 1), family in receipt of social welfare (not in receipt = 0, in receipt = 1). Area level deprivation was assessed in the YWS-NI using the 2017 Northern Ireland Multiple Deprivation Measure (NIMDM; Northern Ireland Statistics and Research Agency; IJepelaar et al., 2017), which assesses deprivation across multiple domains including; (1) income, (2) employment, (3) health and disability, (4) education, skills and training, (5) access to services, (6) living environment, and (7) crime and disorder (Northern Ireland Statistics and Research Agency, 2017). The NIMDM allows for the conversion of household postcodes into Super Output Area data ranked from the most deprived (1) to the least deprived (890) (IJepelaar et al., 2017). For the purposes of the current study, deprivation was ranked in deciles ranging from 1 (high levels of deprivation) to 10 (low levels of deprivation).

#### Parent variables

Parent mental health was measured using the 12-item General Health Questionnaire (GHQ-12; Goldberg & Williams, 1988). The GHQ-12 enquires about the recent (i.e. within the past few weeks) presence of symptoms indicative of psychological distress and poor general functioning. Items are scored on a 4-point scale with scores ranging from 0 (‘better than usual’) to 3 (‘much less than usual’). Total scores ranged from 0 to 12, with scores ≥ 4 indicative of mental health problems. The reliability of the GHQ-12 in the current study was excellent (α = 0.91). Parent ACEs were assessed using the 10-item Adverse Childhood Experiences questionnaire (ACE; Felitti et al., 1998). The ACE questionnaire measures parent’s exposure to ten different childhood adversities including physical and sexual abuse, parental mental health problems, domestic violence and substance abuse in the household. Items are scored dichotomously, with participants responding either yes (1) or no (0). Because four or more ACEs is established as the threshold at which the risk of maladaptive outcomes disproportionately increases (Felitti et al., 1998), parents with ACE scores ≤ 3 were allocated to ‘low ACE score’ and parents with ACE scores ≥ 4 were allocated to ‘high ACE score’.

### Statistical Analysis

In the first stage of data analysis,. descriptive statistics were produced (using IBM SPSS Statistics v.26) to determine the prevalence of exposure to each CT for the overall sample and separately by gender. Following this, chi-square tests of independence were computed between each CT and gender to test for significant gender differences in CT prevalence. Finally, multivariate binary logistic regression models were estimated to determine the effects of child, parent and family predictors on endorsement of each CT, whilst statistically adjusting for the other predictors. Associations are reported as odds ratios (ORs) with 95% confidence intervals.

All subsequent analyses were conducted using Mplus 8.2 (Muthén & Muthén, 2017). Latent class analysis (LCA) was conducted on the binary CATS trauma items, testing models with two to six latent classes. Gender was included as a covariate for all models. Following close examination, the items pertaining to war and natural disaster were removed from the LCA due to both low endorsement (< 5% of sample endorsed these items) and poor univariate entropy, suggesting that these items were not accurate indicators of the latent classes. To determine the optimal number of classes, numerous indices of model fit were inspected including: Bayesian Information Criterion (BIC; Sclove, 1987), sample size adjusted BIC (ssaBIC; Sclove, 1987) and Akaike Information Criterion (AIC; Akaike, 1987). These comparative fit indices assess the improvement of fit of a k-class model to a more parsimonious (k-1 class) model, with smaller values indicating superior model fit. The bootstrap likelihood ratio test (BLRT; McLachlan & Peel, 2000) was also used to compare the improvement in model fit between the k-1 class model and the k-class model. A non-significant value (p ≥ .05) indicates that the more parsimonious k-1 class model should be selected. BIC was given most weight in the current study as it has been shown to be the most reliable indicator for class enumeration (Nylund et al., 2007). Furthermore, entropy, a measure of classification certainty was consulted when determining the optimal solution, with higher values indicating improved classification certainty (Lubke & Muthen, 2007).

Following identification of the best-fitting LCA model, predictors were added to the model using the R3step procedure (Asparouhov & Muthén, 2014), which has been shown to be the superior method of including covariates within LCA (Vermunt, 2010). The covariate analyses were conducted in two stages. Stage one involved regressing class membership on each predictor separately (child variables, family variables, parent variables) to determine the bivariate associations among predictors and latent class membership. The second stage involved including all predictors (child variables, family variables, parent variables) simultaneously to determine the influence of each predictor on class membership, whilst adjusting for the influence of the other predictors. All models were estimated using robust maximum likelihood estimation (Muthén & Muthén, 1998–2018). Listwise deletion was used for missing data for the predictor analyses which is the default when using the R3step procedure.

## Results

### CT prevalence and gender differences in PTE endorsement

Over one-in-three young people aged 11–19 years, (37%, n = 478) experienced at least one CT during their lifetime. Of those trauma-exposed participants, 16.9% (n = 219) reported exposure to one traumatic event, 9.6% (n = 124) reported exposure to two traumatic events, 5.0% (n = 65) reported exposure to three traumatic events and 5.5% (n = 70) reported exposure to four or more traumatic events. The most commonly endorsed CTs were serious accident or injury (18.4%; n = 217), witnessing violence at school or in the community (19.7%; n = 220) and the sudden or violent death of a loved one (11.7%; n = 138). Males were more likely to report serious accident or injury, being threatened, hit or hurt badly in school or the community, witnessing violent, threatening behaviour in school or the community, and being attacked, stabbed, shot at or robbed by threat. Females were more likely to report online sexual harassment compared to males. Table 2 provides a complete overview of the results pertaining to endorsement of each CT and gender differences in endorsement of each CT.

**Table 2:** Experiences of lifetime trauma exposure for total sample, females only and males only

	Total Sample (N = 2199) (%)	Males (n = 662) (%)	Females (n = 631) (%)	${\mathcal{x}}^{2}$
CT1 - Natural Disaster	21 (1.6%)	14 (2.1%)	7 (1.1%)	2.653, p = .156
CT2 - Serious accident or injury	217 (16.8%)	132 (19.9%)	85 (13.5%)	14.885, p = .001
CT3- Threatened, hit or hurt badly in my family	44 (3.4%)	27 (4.1%)	17 (2.7%)	2.791, p = .188
CT4 - Threatened, hit or hurt badly in school or the community	120 (9.3%)	81 (12.2%)	39 (6.2%)*	18.172, p = .005
CT5 - Attacked, stabbed, shot at or robbed by threat	19 (1.5%)	14 (2.1%)	5 (0.8%)	4.675, p = .068
CT6 - Seeing someone in family threatened, hit or hurt badly	85 (6.6%)	43(6.5%)	42 (6.7%)	0.227, p = .847
CT7 - Seeing someone in school or the community threatened, hit or hurt badly	220 (17.0%)	140 (21.1%)	80 (12.7%)*	23.073, p = .000
CT8 - Someone touching my private parts when they shouldn’t. Or making me touch their private parts	25 (1.9%)	10 (1.5%)	15 (2.4%)	0.908, p = .445
CT9 - Someone forcing or pressuring me to do sexual things. Or having to do sexual things when I couldn’t say no	20 (1.5%)	9 (1.4%)	11 (1.7%)	0.178, p = .829
CT10 - Someone asking or pressuring me online to take or send pictures of my private parts, or to touch myself	35 (2.7%)	10 (1.5%)	25 (4.0%)*	6.209, p = .045
CT11 - Someone close to me dying suddenly or violently	138 (10.7%)	67 (10.1%)	71 (11.3%)	0.291, p = .944
CT12 - Stressful or scary medical procedure	55 (4.3%)	28 (4.3%)	27 (4.3%)	0.166, p = .900
CT13 - Being around war	12 (0.9%)	7 (1.1%)	5 (0.8%)	0.427, p = .582
CT14 - Other stressful or scary event	53 (4.1%)	24 (3.6%)	29 (4.6%)	0.434, p = .614

### Multivariate Binary Logistic Regression

The logistic regression model results demonstrated how experiences of out-of-home care significantly increased the odds of endorsing the item ‘threatened, hit or hurt badly in my family’ (OR = 5.38, C.I.= 2.07, 13.99), ‘threatened, hit or hurt badly in school or the community’ (OR = 2.54, C.I. = 1.11, 5.80), ‘seeing someone in family threatened, hit, or hurt badly’ (OR = 3.55, C.I.= 1.53, 8.21), ‘someone touching my private parts when they shouldn’t or making me touch their private parts’ (OR = 4.59, C.I. = 1.19, 17.73), and ‘someone forcing or pressuring me to do sexual things, or having to do sexual things when I couldn’t say no’ (OR = 6.71, C.I.= 1.68, 26.90).

Older age significantly increased the odds of endorsing ‘threatened, hit, or hurt badly in family’ (OR = 1.17, C.I.= 1.02, 1.33), ‘seeing someone in family threatened, hit, or hurt badly’ (OR = 1.53, C.I.= 1.05, 1.27 ), ‘someone asking or pressuring me online to take or send pictures of my private parts, or to touch myself’ (OR = 1.17, C.I.= 1.01, 1.35) and ‘stressful or scary medical procedure’ (OR = 1.13, C.I.= 1.01, 1.27). Young people with families in receipt of social welfare had higher odds of endorsing ‘seeing someone in family threatened, hit, or hurt badly’ (OR = 2.33, C.I. = 1.35, 4.41), ‘seeing someone in school or the community threatened, hit or hurt badly’ (OR = 1.51, C.I. = 1.03, 2.22) and ‘someone forcing or pressuring me to do sexual things, or having to do sexual things when I couldn’t say no’ (OR = 4.25, C.I. = 1.52, 11.91). Living with both biological parents significantly decreased the odds of endorsing ‘seeing some in family threatened, hit, or hurt badly’ (OR = 0.53, C.I. = 0.32, 0.89). Having a parent with mental health difficulties significantly increased the odds of endorsing ‘seeing some in family threatened, hit, or hurt badly’ (OR = 1.81, C.I. = 1.06, 3.09). Individuals who identified as ‘white’ were more likely to endorse the item ‘seeing someone in school or the community threatened, hit or hurt badly’ (OR = 5.49, C.I.= 1.30, 23.18). Finally, having special educational needs increased the odds of endorsing ‘stressful or scary medical procedure’ (OR = 2.51, C.I.=1.25, 5.06).

### Latent Class Analysis

Goodness of fit statistics for the LCA models including gender as a covariate are shown in Table 3. The best log-likelihood failed to replicate for the six-class solution, and therefore, this solution was not considered for the final model. The BIC and ssaBIC values were lowest for the three-class solution compared to all other solutions, whilst LMR-A became non-significant for the four-class solution, suggesting the three-class model to be best-fitting. Furthermore, entropy was highest for the three-class solution, indicating greater classification certainty for the three-class model. Average posterior probabilities for most likely latent class membership indicated that the classes comprising the three-class solution were well-separated (Nylund-Gibson et al., 2018) whilst inspection of profile plots demonstrated that each class comprising the three-class solution represented a distinct sub-group of trauma-exposed young people. Thus, on the basis of model fit and parsimony, the three-class solution was chosen as the final model.

**Table 3:** Model fit statistics for LCA models

Model	Log-likelihood	AIC	BIC	ssa-BIC	Entropy	LMR-A (p)	BLRT (p)
2 classes	-2780.157	5608.315	5730.012	5653.779	0.786	0.0000	0.0000
3 classes	-2730.672	5535.344	5722.961	5605.436	0.825	0.0291	0.0000
4 classes	-2711.111	5522.222	5775.758	5616.941	0.824	0.0676	0.0128
5 classes	-2690.445	5506.891	5826.346	5626.236	0.819	0.1388	0.0000

**Fig. 1:**
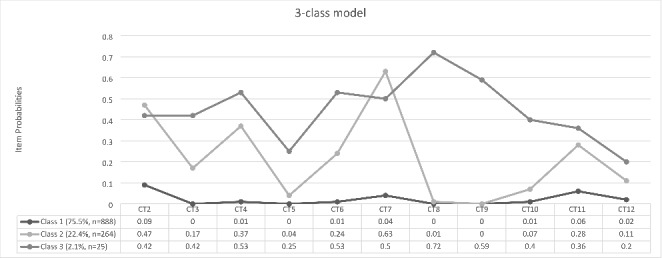
Profile Plot of Childhood Trauma Classes

As illustrated in Fig. 1, class 1 (75.5%, n = 888) comprised the majority of participants and was characterised by low probabilities of endorsing all CTs. Consequently, this class was labelled ‘low exposure’. Class 2 (22.4%, n = 264) was characterised by a relatively high probability of endorsing witnessing violence in the school or community and moderate probabilities of endorsing serious accident or injury and being a victim of violence in school or in the community. Furthermore, this class was characterised by higher probabilities of endorsing any CTs with the exception of sexual assault compared to ‘low exposure’. As a result, this class was labelled ‘moderate-exposure: community-victimization’. Class 3 (2.1%, n = 25) was the smallest class and was characterised by a high probability of endorsing the sexual molestation item ‘someone touching my private parts when they shouldn’t or making me touch their private parts’ and moderate probabilities of endorsing all other CTs, with the exception of ‘attacked, stabbed or robbed by threat’ and ‘stressful or scary medical procedure’. In particular, participants in this class had higher probabilities of endorsing both sexual assault and online sexual harassment, and as such this class was labelled ‘high-exposure: sexual-trauma”.

### Gender effects

Compared to individuals in the ‘low exposure’ class, those in the ‘moderate-exposure: community-victimization’ class were less likely to be female (OR = 0.49, C.I.= 0.33, 0.73), however gender did not significantly predict membership to ‘high-exposure: sexual trauma’ compared to ‘low-exposure’ (OR = 0.92; 95% C.I.=0.33, 2.61). When the reference class was changed to ‘moderate-exposure: community-victimization’ class, participants in ‘low exposure’ were more likely to be female (OR = 2.03; 95% C.I.=1.38, 2.99) however, gender did not significantly predict membership to ‘high exposure - sexual trauma” compared to the ‘moderate-exposure: community victimization’ (OR = 1.87; 95% C.I.=0.59, 5.95).

### Covariate Analyses

For the bivariate analyses (see Table 4), household composition was the only covariate found to significantly predict latent class membership, with individuals in ‘high-risk: sexual trauma’ being significantly less likely to live in a household with both biological parents. Despite all other null effects, inspection of confidence intervals suggested potential significant effects for family in receipt of income or disability benefits which increased risk of membership to ‘high-risk: sexual trauma’ (OR = 3.00; C.I.= 1.29, 6.99), having a parent with mental health difficulties potentially increased risk of membership to ‘moderate-risk: community-victimization’ (OR = 1.56; C.I.=1.04, 2.35) and out-of-home care increased risk of membership to both ‘moderate-risk: community-victimization’ (OR = 2.55; C.I. = 1.05, 5.78) and ‘high-risk: sexual trauma class’ (OR = 8.35; C.I.= 2.54, 27.45).

**Table 4:** Unadjusted Odds Ratios for Child, Family and Parent predictors of Latent Class Membership

Predictor	Class 2: violence OR (95% CI)		Class 3: sexual trauma OR (95% CI)	
Area level deprivation	0.99	(0.93, 1.05)	1.04	(0.89, 1.22)
Age	1.04	(0.97, 1.10)	1.04	(0.87, 1.24)
Family in receipt of income benefits	1.34	(0.94, 1.91)	3.00*	(1.29, 6.99)
Parent Separation (household composition)	0.74	(0.52, 1.06)	0.51*	(0.22, 1.18)
Parent Mental Health (GHQ score)	1.56*	(1.04, 2.35)	1.22	(0.43, 3.49)
Ethnicity	3.32	(0.87, 12.64)	1.39	(0.17, 11.14)
Household employment status	0.90	(0.54, 1.49)	0.67	(0.21, 2.08)
Out-of-home care	2.55*	(1.05, 5.78)	8.35*	(2.54, 27.45)
Special education needs	1.20	(0.69, 2.07)	1.27	(0.35, 4.60)
Parent ACEs	1.19	(0.68, 2.09)	0.87	(0.18, 4.1)
Highest household educational attainment	1.09	(0.75, 1.57)	1.05	(0.4, 2.60)

Note: Class 1 (Baseline) is the reference category, * - significant at p < .05

Despite the null effects observed in the bivariate analyses, multivariate analyses were then conducted using the R3step procedure to determine the covariates which significantly predicted latent class membership, whilst statistically controlling for all variables (see Table 5). Surprisingly, when compared to the reference class, no covariate was found to significantly predict membership to either ‘moderate-risk: community-victimization’ nor ‘high-risk: sexual trauma’. However, inspection of confidence intervals for the odds ratios suggested that family in receipt of income or disability benefits (OR = 4.69; C.I.= 1.35, 16.28) and out-of-home care experience (OR = 10.36; C.I.= 3.04, 35.37) increased risk of membership to the ‘high-risk: sexual trauma’ compared to the reference class. Notably, when the reference class was changed to ‘high-risk: sexual trauma class’, individuals in ‘low-risk’ were less likely to have parents in receipt of income or disability benefits (OR = 0.21; C.I.=0.06, 0.74) and less likely to have been in out-of-home care (OR = 0.10; C.I.= 0.03, 0.33). Likewise, compared to ‘high-risk: sexual trauma class’, individuals in ‘moderate-risk: community victimization’ were less likely to have family in receipt of income or disability benefits (OR = 0.27; C.I.= 0.07, 1.00) and were less likely to be in out-of-home care (OR = 0.24, C.I.= 0.06, 0.94).

**Table 5:** Adjusted Odds Ratios for Child, Family and Parent predictors of Latent Class Membership

Predictor	Class 2: violence OR (95% CI)		Class 3: sexual trauma OR (95% CI)	
Area level deprivation	0.99	(0.92, 1.06)	1.13	(0.95, 1.35)
Age	1.05	(0.98, 1.12)	1.04	(0.86, 1.26)
Family in receipt of income benefits	1.26	(0.80, 1.99)	4.69*	(1.35, 16.28)
Parent Separation (household composition)	0.78	(0.51, 1.20)	0.87	(0.30, 2.49)
Parent Mental Health (GHQ score)	1.44	(0.93, 2.25)	1.14	(0.35, 3.71)
Ethnicity	2.27	(0.63, 8.27)	1.71	(0.09, 31.83)
Household employment status	1.22	(0.64, 2.35)	1.18	(0.36, 8.83)
Out-of-home care	2.50	(0.99, 6.30)	10.36*	(3.04, 35.37)
Special education needs	1.10	(0.62, 1.95)	0.91	(0.23, 3.50)
Parent ACEs	0.95	(0.51, 1.74)	0.78	(0.15, 3.96)
Highest household educational attainment	1.33	(0.86, 2.01)	1.39	(0.39, 5.02)

Note: Class 1 (Baseline) is the reference category, * - significant at p < .05

*CT2 = serious accident or injury, CT3 = threatened, hit or hurt badly in family, CT3 = threatened, hit or hurt badly in school or the community, CT5 = attacked, stabbed, shot at or robbed by threat, CT6 = seeing someone in family threatened, hit or hurt badly, CT7 = seeing someone in school or the community threatened, hit or hurt badly, CT8 = sexual molestation, CT9 = sexual assault, CT10 = online sexual harassment, CT11 = sudden or violent death of loved one, CT12 = stressful or scary medical procedure*

## Discussion

Results showed that in the NI population, almost four in ten young people reported exposure to at least one CT during their lifetime, with a sizeable proportion also reporting exposure to multiple CT’s. Compared to the previously summarised nationally representative investigations of CT prevalence in young people such as those conducted in the US (e.g. Copeland et al., 2007; McLaughlin et al., 2013) and Denmark (e.g. Landolt et al., 2013), the prevalence of CT exposure in NI was considerably lower. Comparing trauma prevalence estimates across countries is laden with challenges especially given the considerable heterogeneity in the operationalisation of what constitutes a traumatic stressor and the resulting types of traumatic events accessed across individual studies (de Vries & Olff, 2009). In the current study, the Child and Adolescent Trauma Screener (CATS) was employed to assess CT exposure, a checklist which comprised of traumatic events which align with both the DSM-5 and ICD-11 definitions of a traumatic stressor (Sascher et al., 2017). Conversely, other studies have relied on instruments which conformed with the DSM-IV A1 criterion (e.g. Copeland et al., 2007; Landolt et al., 2013; McLaughlin et al., 2013), of which several traumas such as the non-violent death of a loved one and serious illness of a loved one, have been subsequently removed (Kilpatrick et al., 2013). Likewise, the number of CTs assessed across studies differs significantly such that prior studies (i.e., Copeland et al., 2007; McLaughlin et al., 2013) have included more CTs than the present study, and thus, this is likely to have influenced prevalence rates. Furthermore, CT exposure prevalence estimates may differ depending on cultural and political factors (Atwoli et al., 2015), as well as geographical location, sociodemographic characteristics and the individual trauma histories of the population under investigation (Benjet et al., 2016). For instance, Viola et al. (2016) demonstrated how the prevalence of CT is significantly higher in North America compared to the UK, and thus, this may explain why the prevalence of CT was lower in the current study compared to those conducted in the US. Finally, research has shown how screening methods can influence prevalence estimates such that self-report questionnaires (as was used in the current study) generate lower prevalence estimates than personal interviews (Saunders et al., 2014), with some prior representative surveys of young people using the latter (e.g., Copeland et al., 2007; McLaughlin et al., 2013). Thus, it is clear that there are multiple potential explanations for the significant heterogeneity rates of CT prevalence within the literature, and thus, these should be taken into consideration when comparing estimates across studies.

Notably, the prevalence of CT exposure in young people in the current study is higher than the trauma-exposure prevalence of 31.1% reported in England and Wales (Lewis et al., 2019), indicating CT exposure to be more prevalent among NI youths compared to other UK nations. There are a myriad of potential explanations for this finding, most prominent being the impact of the “Troubles”. The continued effects of the “Troubles” which include marginalisation, socio-economic adversity, social deprivation as well as intermittent instances of inter-community violence (Browne & Dywer, 2014), may cultivate a developmental environment in which CT exposure is more probable. NI is also one of the most socio-economically deprived areas within the UK (Abel et al., 2016), with socio-economic deprivation known to increase vulnerability to experiencing a broad range of stressors across multiple levels of a young person’s ecology (Evans & Kim 2013). Other potential explanations may be that parental trauma stemming from the “Troubles” in NI may be linked to the higher prevalence of CT exposure in young people; prior research has demonstrated a link between parental trauma and heightened risk of offspring trauma exposure and distress (e.g. Cross et al., 2018; Zerach et al., 2016). However, the current study provided no such evidence of a transgenerational effect, and thus, detailed investigations of whether parental trauma stemming from the “Troubles” increases risk for CT is required in future studies. Alternatively, the higher prevalence rate observed in the present study may be consequential of the methodological procedure adopted such that CT exposure was assessed in young people of various ages, whereas Lewis et al. (2019) assessed trauma exposure at 18 years. A detailed exploration of factors relating to the higher levels of CT exposure in NI compared to other areas within the UK is warranted in future research.

This study found that males were more likely to report traumatic exposures of a violent nature, a finding which aligns with much of the trauma literature (Tolin & Foa, 2008). Moreover, consistent with prior studies (Zetterström Dahlqvist & Gillander Gådin, 2018; Stahl & Dennhag, 2020; Zelviene et al., 2020), results demonstrated that females were more likely to report online sexual harassment, further evidencing how online sexual harassment is becoming a particularly common experience for young females (Zetterström Dahlqvist & Gillander Gådin, 2018). Notably, contrary to previous research where sexual traumas were much more prevalent for females compared to males (e.g. Finkelhor et al., 2014), no such gender differences in either sexual molestation or sexual assault were identified in the present study, although females did endorse those traumas to a greater extent than males. The relatively low endorsement of the sexual trauma items in the present study may explain this finding. Compared to other large-scale investigations of sexual trauma prevalence such as the Sexual Abuse and Violence in Ireland study (SAVI; McGee et al., 2002), which found that almost one third of females and a quarter of males in the Irish adult general population reported some form of childhood sexual abuse, the prevalence rates in the current study are markedly lower. The particularly sensitive nature of these items may have precluded a young person from divulging such traumatic experiences with factors such as feelings of shame, guilt and fear of perpetrator identified as primary barriers to disclosure in young people (Münzer et al., 2016). Conversely, the SAVI study was extensive in the examination of childhood sexual trauma, ensuring that items were precisely defined and that various forms of childhood sexual trauma including ‘non-contact’, ‘non-penetrative contact’ and ‘penetrative’ abuse were captured (McGee et al., 2002). The type of instrument utilised and the phrasing of questions asked have been identified as being influential in the self-reporting of sexual trauma (Abbey et al., 2005; Stoltenborgh et al., 2011). Thus, the comparatively vague phrasing of the sexual trauma items in the current study may explain their low endorsement. These findings would suggest that a general mental health survey may not be the most appropriate vehicle through which to assess histories of sexual trauma in young people and that a dedicated study akin to the SAVI study may be necessary to deliver robust prevalence rates of childhood sexual trauma in the NI population.

Confirming both previous research (O’Donnell et al., 2017) and the initial hypothesis that a trauma group characterised by low levels and another characterised by high levels of trauma exposure would be identified, LCA results provided strong support for three distinct trauma classes ;‘low-exposure’, ‘moderate-exposure: community-victimization’ and ‘high-exposure: sexual-trauma’. In the current study, 63% of young people were labelled as ‘low-exposure’, consistent with previous research which has indicated that the vast majority of individuals in the general-population are relatively unaffected by trauma exposure (e.g. Liang et al., 2020; McChesney et al., 2015; Shevlin & Elklit, 2008; Houston et al., 2011; McAnee et al., 2019; Haahr-Pedersen et al., 2020; Contractor et al., 2018). Similar to the ‘interpersonal non-sexual trauma’ class identified by Ford et al. (2010), and the ‘interpersonal non-sexual class’ identified by McChesney et al. (2015), the second largest class, which was deemed quantitatively rather than qualitatively different to ‘low risk’, was ‘moderate-exposure: community-victimization’. This class captured those individuals exposed to high levels of witnessing violence in the school or community and moderate levels of exposure to serious accident or injury and being themselves victims of violence in school or the community. The identification of the ‘moderate-risk: community-victimization” class in the current study highlights how a relatively large proportion of young people in the NI general-population have experienced multiple co-occurring traumas of a violent nature.

Consistent with previous research (McChesney et al., 2015; Liang et al., 2020; Shevlin & Elklit, 2008), the least populated CT class, which comprised only 2% of the sample, contained those young people who had experienced high levels of exposure to different kinds of ‘high-impact’ CTs. This class was deemed both quantitatively and qualitatively different to ‘low-risk’ and ‘moderate-risk: community victimization’. Sexual molestation was a particularly pertinent trauma for this sub-group of participants, with sexual assault and online sexual harassment also heavily endorsed. The high levels of co-occurrence among the sexual trauma events is unsurprising given that sexual-related traumas are highly interrelated with young people exposed to sexual-abuse being particularly vulnerable to subsequent victimizations of a similar nature (Schouwenaars et al., 2016; Villalta et al., 2020). Likewise, the co-occurrence among the majority of trauma types supports previous research whereby exposure to most forms of victimization or traumatic experiences increases the likelihood that a young person will be exposed to additional traumas (Finkelhor et al., 2007b). The identification of this trauma class highlights a small cluster of young people in the NI general-population who have been exposed to multiple kinds of co-occurring CTs, particularly those of a sexual nature.

Findings surrounding the role of gender in distinguishing CT exposure groups partially supported the original hypotheses. Consistent with the initial hypothesis, results showed how males were more likely to be in ‘moderate-risk: community-victimization’. Surprisingly, in contradiction to the original hypothesis, being female did not significantly predict membership to ‘high-risk: sexual-trauma’. However, a prior LCA study conducted on a sample of Greenlandic adolescents (Karsberg et al., 2014) also found that gender did not predict membership to the sexual trauma class, thus it is possible that in some populations female gender may not serve to distinguish a trauma group characterised by high risk of exposure to various CT types from other groups. On the other hand, this may again be reflective of the low endorsement of sexual trauma items in the present study, which may have led to difficulty in detecting statistically significant effects.

Findings from the predictor analyses of latent class membership did not align with the earlier hypotheses as the only variable which significantly predicted latent class membership within the bivariate analyses was household composition. Mirroring findings from previous research (e.g. Shevlin & Elklit, 2008; McChesney et al., 2015; McLaughlin et al., 2013), individuals in ‘high-risk: sexual-trauma’ were more likely to live with less than both biological parents, suggesting that young people living in fragmented households are at greater risk of exposure to more severe and multiple types of CTs. Surprisingly, no variables were found to differentiate ‘low-risk’ from the other trauma classes within the multivariate analyses. Similar to the bivariate analyses, the null effects observed multivariate analyses may be due to the uneven distribution of participants across the latent trauma classes which may have resulted in insufficient statistical power to detect significant effects. Despite this, our findings suggest that families in receipt of social welfare and experiences of out-of-home care were potentially important in increasing risk of membership of ‘high-risk: sexual-trauma’. It is unsurprising that experiences of out-of-home care emerged as a potential risk factor for membership of the most severe trauma class, especially given a recent study reported how 70% of young people in out-of-home-care reported exposure to a ‘Criterion A’ traumatic stressor (Hiller et al., 2021). Furthermore, the potential role of family being in receipt of social welfare is expected given that this is considered an index of socio-economic status which has frequently been linked to an increased risk of trauma exposure for young people (e.g. Brattström et al., 2015; McLaughlin et al., 2013; Coulton et al., 2007; Reiss et al., 2019).

Parental variables also failed to significantly differentiate the CT exposure groups, however, the potential distinguishing role of parental factors was investigated only in an exploratory manner. Nevertheless, through examining the influence of parental variables on individual CTs, having a parent with mental health difficulties increased risk of endorsing witnessing violence in the family and online sexual harassment. Previous research has shown how poorer maternal emotional wellbeing increases the likelihood for a child of witnessing domestic violence (Meltzer et al., 2009), thus one explanation may be that poor parent mental health increases vulnerability to domestic violence exposure which in turn places a young person at greater risk of witnessing violence in the home. With regard to increased risk of online sexual harassment, research has shown that maternal psychopathology may result in inadequate monitoring of a young person which in turn may lead to risky sexual behaviors (Hadley et al., 2011; Ryan et al., 2015). It is possible that parental psychopathology may increase a young person’s risk to online sexual harassment via poorer monitoring of the young person and their online activity. Further research is necessary to determine the mechanisms underpinning the association between parental psychopathology and increased risk of child exposure to CTs, particularly in relation to paternal psychopathology.

There are several notable strengths of the current study. The investigation of a large nationally representative sample of young people provides a comprehensive understanding of the prevalence, patterns and correlates of trauma-exposure in the NI general-population. However, despite the strengths of the present study, it is important that the findings are considered in light of several limitations. Firstly, the use of a general-population sample of young people limits generalisability of findings to clinical samples where CT exposure prevalence may greatly exceed those observed in the present study (Saunders & Adams, 2014). Furthermore, the use of self-report measures to assess exposure to CTs in the present study may have led to biased responses from participants (Kreitchmann et al., 2019). Given that unequal class sizes can lead to decreased statistical power (Tekle et al., 2016) and smaller class sizes can increase standard errors which can obscure significant associations (Houston et al., 2011), the unequal distribution of participants across the latent classes may have resulted in insufficient power to detect significant effects for child, familial and parental risk factors of the latent classes. Nevertheless, inspection of confidence intervals provided useful information as to those factors which may place an individual at greater risk of membership to classes characterised by higher trauma exposure levels and complex trauma patterns. Moreover, the LCA approach to investigating trauma is not without its limitations, most notably being that these classes do not inform us about the trauma age of onset, frequency, duration, severity or the perpetrator, all of which are also influential elements of the traumatic experience. Finally, the cross-sectional nature of the current study prohibits inferences regarding causality to be made.

The overall aim of the present study was to determine the prevalence, patterns and correlates of CT exposure using data from the first-ever representative survey of CT exposure in young people living in NI. The current study adds to a small repertoire of representative studies investigating the prevalence of CT exposure in young people and thus, assists in illuminating how CT differs across different countries and cultural contexts. In keeping with the extant evidence base, findings from the present study support the idea that trauma exposure is relatively commonplace in the lives of many young people. Moreover, this study was first to examine the co-occurrence of CT among young people in Northern Ireland and the wider UK context. Findings demonstrate that distinct groups of young people with similar patterns of CT are clearly identifiable within the NI context. Specifically, the identification of three discrete trauma classes which collectively summarize the distribution of trauma-exposure among young people in NI, provides important insights into the variations of trauma-exposure levels across the NI population as well as how trauma types co-occur and cluster together. Moreover, the results of the present study solidify the importance of comprehensive trauma screening to determine the full spectrum of CTs to which a young person has been exposed. Furthermore, the finding that males were likelier to report trauma patterns characterised by community violence as well as the potential role of out-of-home care experiences and family in receipt of social welfare in predicting membership to trauma groups characterised by moderate to high levels of CT exposure, highlights risk-factors which increase a young person’s vulnerability to more complex trauma profiles, a finding which will be of interest for clinicians and policy-makers alike.
